# *Fusarium sacchari* Effector FsMEP1 Contributes to Virulence by Disturbing Localization of Thiamine Thiazole Synthase ScTHI2 from Sugarcane

**DOI:** 10.3390/ijms252212075

**Published:** 2024-11-10

**Authors:** Lulu Wang, Deng Wu, Tianshu Hong, Qianqian Ren, Shichao Wang, Yixue Bao, Wei Yao, Muqing Zhang, Qin Hu

**Affiliations:** 1 Guangxi Key Laboratory of Sugarcane Biology, Nanning 530004, China; 18302385802@163.com (L.W.); rq130084@163.com (Q.R.); yaoweimail@163.com (W.Y.);; 2 State Key Laboratory for Conservation and Utilization of Subtropical Agro-Bioresources, Guangxi University, Nanning 530004, China; 3 College of Agronomy, Guangxi University, Nanning 530004, China

**Keywords:** sugarcane, *Fusarium sacchari*, metalloproteinase, plant immunity

## Abstract

*Fusarium sacchari* is a significant pathogenic fungus that causes sugarcane Pokkah Boeng. Proteins secreted by pathogenic fungi can be delivered into hosts to suppress plant immunity and establish infection. However, there is still much to be discovered regarding *F. sacchari*’s secreted effectors in overcoming plant immunity. In this paper, we characterize a novel effector called FsMEP1, which is essential for the virulence of *F. sacchari*. FsMEP1 contains a conserved zinc-binding motif sequence, HEXXH, and is highly expressed during host infection. Using the *Agrobacterium tumefaciens*-mediated transient expression system, it was confirmed that FsMEP1 could suppress Bcl-2-associated X protein (BAX)-triggered cell death, callose deposition, and ROS explosion in *Nicotiana benthamiana*. Furthermore, the deletion of FsMEP1 demonstrated its requirement for contributing to the pathogenicity of *F. sacchari* in sugarcane. Further analysis revealed that FsMEP1 could interact with the sugarcane thiamine thiazole synthase ScTHI2 and disrupt its normal localization, thereby inhibiting the synthesis of thiamine and the defense responses mediated by ScTHI2. Based on these findings, we propose that ScTHI2 represents a potential molecular target for improving sugarcane resistance to Pokkah Boeng disease.

## 1. Introduction

Sugarcane is an important global sugar crop and industrial raw material, accounting for more than 85% of the total sugar in the world, and has been successfully commercialized in more than 100 countries [1]. Pokkah Boeng disease (PBD), caused by the *Fusarium fujikuroi* species complex (FFSC), was first identified in Java in 1890. The term “Pokkah Boeng” originates from Javanese and refers to a malformed apex. PBD is one of the most destructive fungal diseases of sugarcane production [2,3]. *Fusarium sacchari*, a species of the FFSC, is the most common and widely distributed causative agent of PBD in China and leads to great losses in the sugarcane yield and quality by interfering with leaf photosynthesis and stem tip growth [4,5]. Currently, planting resistant varieties is the most effective strategy to control PBD. However, research on the pathogenesis mechanisms of *F. sacchari* and the interaction between sugarcane and *F. sacchari* is still lacking. This limitation hinders the application of modern molecular breeding technology for improving PBD resistance.

Research concentrating on plants under causative agent attack have improved our comprehension of plant–pathogen interactions [6]. In most cases, pathogens exploit specialized characteristics to set up a parasitic relationship with their host plants. In order to effectively defend themselves against pathogen attacks, plants have evolved a two-tiered immune system known as pattern-triggered immunity (PTI) and effector-triggered immunity (ETI) [7]. PTI relies on the recognition of the common features of microbial pathogens known as pathogen-associated molecular patterns (PAMPs) by diverse members of pattern-recognition receptors (PRRs) [8,9]. To break through the host’s PTI, successful pathogens have developed effector molecules, either by inhibiting PTI signaling or by preventing the host detection of their PAMPs [10]. For example, the alkaline protease (AprA) of *Pseudomonas aeruginosa* acts as a TLR5 and FLS2 signaling inhibitor, escaping recognition from the congenital immune systems of plants [11]. Effector protein Ecp6 of *Cladosporium fulvum* prevents host immune activation by sequestering the chitin oligosaccharides released from the cell walls [12]. In the long-term evolution between hosts and pathogens, some plants have acquired resistance (R) proteins that recognize these varied effector molecules, triggering a secondary immune response (ETI) [13]. This process depends on intracellular nucleotide-binding leucine-rich repeat (NLR) proteins [14], which can detect effector proteins and activate the hypersensitive response (HR) [15]. Therefore, exploring pathogenic effectors and their targets is crucial for improving plant resistance.

Metalloproteinases (MEPs) are a class of proteinases whose active centers depend on metal ions, most of which contain Zn^2+^ in the active sites [16]. Most zinc metalloproteinases have a conserved zinc-binding motif sequence, HEXXH, in which catalyzed zinc ions are chelated by two histidine residues [17]. MEPs have been previously associated with virulence in both fungi and bacteria. For instance, a fungalysin metalloprotease gene known as *Cgfl* was identified from *Colletotrichum graminicola*, which could enhance virulence on maize leaves and roots while simultaneously inhibiting leaf chitinase activity, thereby promoting disease development. [18]. Avr-Pita, an MEP effector from *Magnaporthe oryzae*, binds directly to the Pi-ta LRD region inside the plant cell to initiate the Pita-mediated defense response. Meanwhile, abundant Avr-Pita varieties have been evolved in *M. oryzae* to evade surveillance from Pita and intercept the host immunity signaling cascade [19,20,21]. Taken together, the contribution of MEPs to pathogenicity suggests that they play a vital role in infection and disease spreading.

Thiamine (vitamin B1, VB1) is a crucial component in numerous enzymatic reactions for all living organisms [22]. Animals obtain VB1 from their diets, while plants, fungi, and bacteria must obtain it by de novo synthesis [23]. The VB1 biosynthesis in plants is roughly similar to that in prokaryotes, involving the synthesis of a thiazole moiety and a pyrimidine moiety, both of which occur in chloroplasts [24]. In plants, thiamine is formed by bridging a thiazole (4-amino-2-methyl-5-hydroxymethylpyrimidine phosphate, HET-P) ring with a pyrimidine (4-amino-5-hydroxymethyl-2-methylpyrimidine phosphate, HMP-P) ring [25]. HEP-T synthase (THI1) and THI2 (ortholog of THI1) catalyze the biosynthesis of the thiazole moiety [26], and HMP-P synthase (THIC) catalyzes the biosynthesis of the pyrimidine moiety [27,28]. Recent research has revealed that thiamine not only plays an important role in plant growth and development but also significantly influences the competition between plants and pathogens. Studies have shown that the necrotic lesions caused by *Magaporthe grisea* on rice, the typical blight symptoms caused by *Xanthomonas oryzae* pv *oryzae* on rice, and the mottling symptoms prompted by *Pepper mild mottle virus* on tobacco were significantly suppressed in plants sprayed with exogenous thiamine, suggesting that thiamine protects plants from a wide range of fungi, bacteria, and pathogens [29]. Furthermore, THI1/THI2 are the essential enzymes in thiamine biosynthesis. The biosynthesis of VB1 is involved in plant responses to abiotic and biological stresses [30]. In *Arabidopsis*, the expression level of *AtTHI1* was upregulated by various abiotic stresses, such as osmotic, salinity and oxidative stresses. Further analysis proved that AtTHI1 participated in manipulating ABA-induced stomatal closure by interacting with Ca^2+^-dependent protein kinase at the plasma membrane [30]. In wheat, Chinese wheat mosaic virus (CWMV) could direct the chloroplast-localized TaTHI2 to the wheat cell membrane, and the overexpression of *TaTHI2* in the wheat conferred enhanced resistance to CWMV with accumulated ROS production by suppressing TaCPK5-activated TaCAT1 [31]. Notably, THI1/THI2 in plants represent a valuable gene that can balance abiotic and biotic stress tolerance, but the regulatory mechanisms of THI1/THI2 remain largely unknown.

In this study, we concentrated on the functional analysis of the FsMEP1 in *F. sacchari*–sugarcane interactions. We discovered that FsMEP1 has secretory activity and can be localized to the *Nicotiana benthamiana*’s nucleus to inhibit the immunity responses, and the knocking out of FsMEP1 significantly reduces the pathogenicity of *F. sacchari* on sugarcane. Further experiments demonstrated that FsMEP1 could interact with the sugarcane thiamine thiazole synthase ScTHI2 and disturb its normal localization, thereby blocking up the synthesis of VB1 and the defense responses that are mediated by ScTHI2. Our results provide new insights into the function of MEP effectors in pathogen–host interactions, as well as new clues, such as the application of the host-induced gene silencing (HIGS) of FsMEP1 and the manipulation of endogenous VBI synthesis by the overexpression of ScTHI2, to improve the resistance of sugarcane to PBD.

## 2. Results

### 2.1. Identification and Characteristic Analysis of FsMEP1

**Figure 1:**
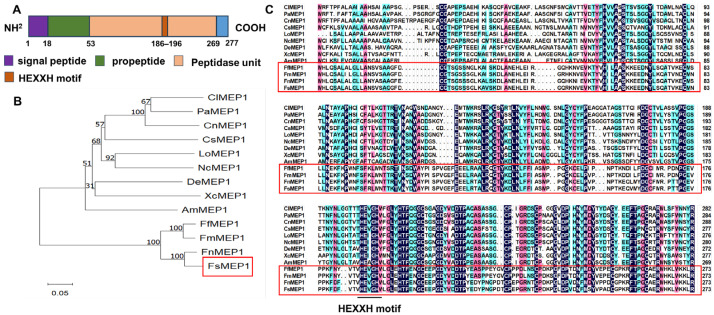
Identification and characterization of FsMEP1 protein. (**A**) Schematic view of FsMEP1 protein domains. (**B**) Phylogenetic analysis of FsMEP1 and metalloproteases from other fungi using the neighbor-joining method in MEGA11 software, with the bootstrap repetitions set to 1000 in this method. Cl, *Chaetomidium leptoderma* (KAK4151148.1); Pa, *Parathielavia appendiculata* (KAG7053257.1); Cn, *Corynascus novoguineensis* (KAK4247909.1); Cs, *Colletotrichum scovillei* (KAG7053257.1); Lo, *Lasiosphaeria ovina* (KAK3365578.1); Nc, *Neurospora crassa* (XP_957425.1); De, *Diaporthe eres* (KAI7780499.1); Xc, *Xylaria cubensis* (KAI0858187.1); Am, *Apiospora marii* (KAK7923673.1); Ff, *Fusarium fujikuroi* (KLP05595.1); Fm, *Fusarium mexicanum* (KAF5531805.1); Fn, *Fusarium napiforme* (KAF5561731.1); Fs, *F. sacchari*. (**C**) Multiple sequence alignment of FsMEP1 and its homologues. Red frames indicated the MEP proteins from the genus *Fusarium*.

Our previous studies completed the prediction of effectors in *Fusarium sacchari* [32], and among these putative effectors, a gene encoding an extracellular protein with a typical zinc-dependent metalloprotease domain was cloned from the cDNA of *F. sacchari* and named FsMEP1. The encoding sequence of FsMEP1 is composed of 834 nucleotides and encodes a protein of 277 amino acids with an N-terminal signal peptide (1–18 aa) and a pappalysin M43 peptidase domain within the zinc-dependent metalloproteinase region (53–269 aa), and it harbors the typical HEXXH motif (186–196 aa) (Figure 1A). To investigate the evolutionary relationship between FsMEP1 and other MEP proteins from fungal species, a phylogenetic tree was constructed using the neighbor-joining method. The results showed that the FsMEP1 was closely related to the MEP proteins from *Fusarium fujikuroi* (FfMEP1), *Fusarium mexicanum* (FmMEP1), and *Fusarium napiforme* (FnMEP1), which are different species of the genus *Fusarium* (Figure 1B). Protein sequence alignment analysis revealed that MEP proteins are highly conserved in different *Fusarium* species, implying that they may have similar functions (Figure 1C).

### 2.2. FsMEP1 Was Upregulated in Invasion Stage and Mainly Localized in Host Nucleus

**Figure 2:**
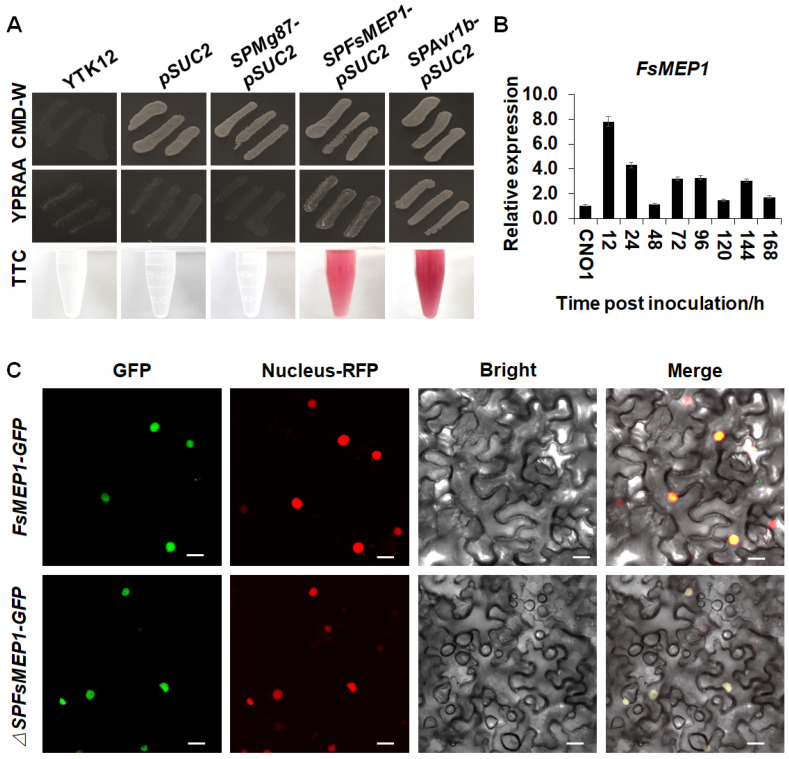
Signal peptide secretory activity verification and subcellular localization analysis of FsMEP1. (**A**) Secretory function verification of FsMEP1 signal peptide in yeast strain YTK12. CMD-W is a complete media used to select yeast strain YTK12 carrying different pSUC2 constructions. YPRAA media, a yeast peptone raffinose with antimycin medium, was used to indicate invertase secretion. YTK12 carrying the SPAvr1b-pSUC2 vector was used as a positive control, and YTK12 carrying the SPMg87-pSUC2 vector or pSUC2 empty vector was used as a negative control. The growth on YPRAA medium and change in color of TTC confirmed the invertase secretion. Representative photographs are shown. (**B**) The induced expression pattern of *FsMEP1* in the interaction between *F. sacchari* and sugarcane. Values are the means ± SD; *n* = 3. (**C**) Subcellular localization analysis of FsMEP1 and ΔSPFsMEP1 in *N. benthamiana* leaves. Representative leaves were taken at 48 h post-inoculation. Bar: 20 μm.

As a typical small, cysteine-rich protein (SCP), *FsMEP1* was also predicted to contain a signal peptide in its N-terminal. To confirm the secretory activity of this signal peptide, the yeast signal sequence trap system was performed and further confirmed by 2,3,5-triphenyltetrazolium chloride (TTC) catalysis detection. The results showed that the signal peptide of *FsMEP1* (SPFsMEP1) could mediate the secretion of invertase, thereby enabling the yeast strain YTK12 carrying *SPFsMEP1-pSUC2* to grow normally on the medium with raffinose as the sole carbon source and the catalyzation of TTC into triphenylformazan, resulting in a red color reaction (Figure 2A). These results suggested that the signal peptide of FsMEP1 is functional and probably secreted by *F. sacchari*. To further study the function of FsMEP1 in the interaction between *F. sacchari* and sugarcane, we examined the expression pattern of *FsMEP1* in sugarcane leaves that were inoculated with *F. sacchari*, and we observed that the expression level of FsMEP1 was induced nearly eight times more in the sugarcane leaves at 12 h post-inoculation (hpi) with *F. sacchari* than that in mycelia and then gradually decreased, which suggested that FsMEP1 may be involved in the pathogenicity of *F. sacchari* towards sugarcane (Figure 2B). We further confirmed that FsMEP1 was localized to the nuclei of *Nicotiana benthamiana* epidermal cells by merging green fluorescent protein (FsMEP1-GFP) together with its C-terminus, and the removal of the FsMEP1 signal peptide (ΔSPFsMEP1-GFP) did not affect its subcellular localization (Figure 2C).

### 2.3. FsMEP1 Contributed to the Pathogenicity of Fusarium sacchari

**Figure 3:**
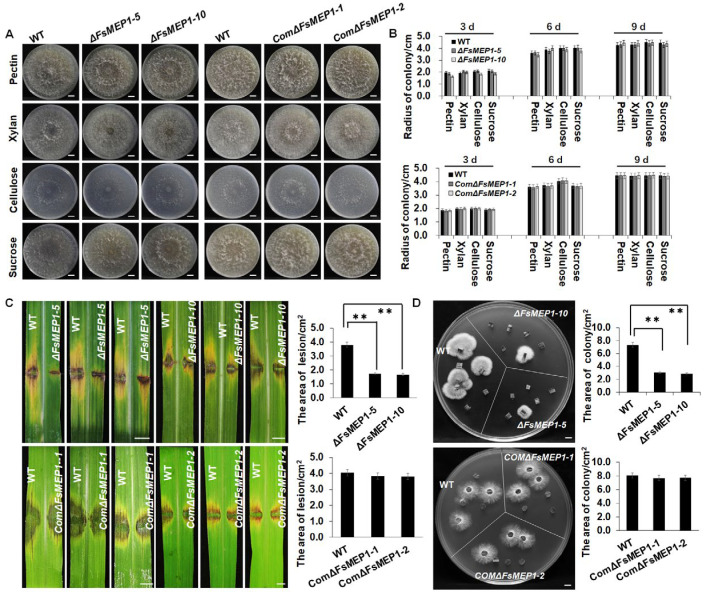
FsMEP1 was not required for *F. sacchari* growth and conidium formation but contributed to its pathogenicity. (**A**) The growth phenotypes of *ΔFsMEP1*, *ComΔFsMEP1*, and wild-type strains grown on medium with pectin, xylan, cellulose, or sucrose as carbon source for 9 days. (**B**) Statistical analysis of the vegetative growth rate of *ΔFsMEP1*, *ComΔFsMEP1*, and wild-type strains grown on medium with pectin, xylan, cellulose, or sucrose as carbon source. Values are the means ± SD; *n* = 6. (**C**) The pathogenicity of ΔFsMEP1, ComΔFsMEP1, and wild-type strains on sugarcane leaves. Representative leaves were photographed at 7 d post-inoculation. Values are the means ± SD; *n* = 6. (**D**) The growth of hyphae recovered from the infected leaves of *ΔFsMEP1*, *ComΔFsMEP1*, and wild-type strains on PDA medium. Representative photographs were taken at 5 d. Values are the means ± SD; *n* = 6. Statistical analyses were performed using Student’s *t* test; ** represent significant differences at *p* < 0.01. Bar: 1 cm.

To establish whether FsMEP1 is involved in the mycelial growth or pathogenicity of *F. sacchari*, *FsMEP1* knockout mutants (*ΔFsMEP1*) and complementary mutants (*Com-ΔFsMEP1*) were generated by homologous recombination through *Agrobacterium tumefaciens*-mediated transformation. The schematic diagrams for the gene deletion and gene insertion are shown in Figure S1A,B, and the positive strains were examined by PCR detection (Figure S1C,D). The knockout mutants *ΔFsMEP1*-5 and *ΔFsMEP1*-10 and the complementary mutants *Com-ΔFsMEP1-1* and *Com-ΔFsMEP1-2* were selected for further research (Figure S1C,D). The growth rate of the colony, spore morphology, and spore number of *ΔFsMEP1* and *Com-ΔFsMEP1* on different carbon source media were detected to evaluate the roles of FsMEP1 in *F. sacchari* growth and development. There was no statistical difference in the colony growth rate, spore morphology, or spore number between the *F. sacchari* wild-strain CNO-1 and FsMEP1 mutants, indicating that FsMEP1 did not participate in the fungal growth and development (Figure 3A,B and Figure S2). Further, we tested the pathogenicity of the FsMEP1 mutants on sugarcane leaves and the lesion area caused by the WT, and the *ΔFsMEP1* and *Com-ΔFsMEP1* strains were analyzed to evaluate the roles of FsMEP1 in *F. sacchari* pathogenicity. Significantly, the necrotic lesions on the leaves inoculated with ΔFsMEP1 were smaller than those induced by the WT, and *Com-ΔFsMEP1* restored the virulence of *ΔFsMEP1* at 120 h post-inoculation (Figure 3C). Furthermore, less fungal biomass was found in the leaves that were inoculated with *ΔFsMEP1* than that of the WT and *Com-ΔFsMEP1* in the fungal recovery assays (Figure 3D). In all, these results confirmed that FsMEP1 contributed to the pathogenicity of *F. sacchari*, and that this might be the result of FsMEP1 interacting with the host rather than affecting the fugal growth.

### 2.4. FsMEP1 Inhibited Plant Immunity Responses and Enhanced Host Susceptibility

**Figure 4:**
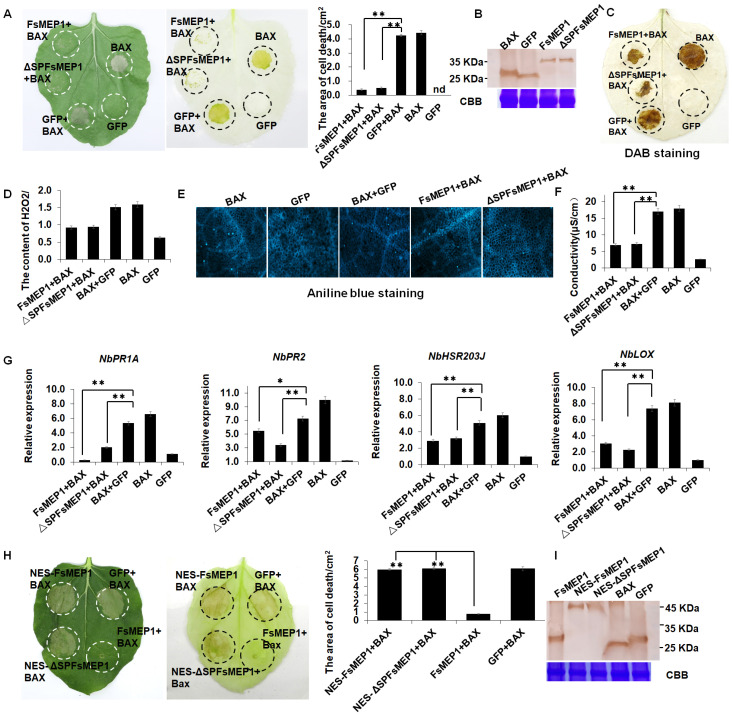
FsMEP1 could suppress the cell death cause by BAX in *Nicotiana benthamiana.* (**A**) *N. benthamiana* leaves were separately infiltrated with the indicated *A. tumefaciens* suspensions to detect the role of FsMEP1 in manipulating plant immune responses. GFP and BAX served as negative and positive controls, respectively. The cell death areas of the indicated infiltrated regions were calculated at 5 d post-inoculation. Values are the means ± SD; *n* = 12. (**B**) The efficiency of the translation of the indicated proteins was determined by Western blotting using HA antibody. The Coomassie bright blue-stained Rubisco protein is shown as the total protein loading control. (**C**,**D**) H_2_O_2_ staining and determination. Values are the means ± SD; *n* = 6. (**E**,**F**) Callose staining and determination. Values are the means ± SD; *n* = 6. (**G**) Relative expression assay of defense-related genes in *N. benthamiane* leaves and the transient expressions of the indicated constructs. Values are the means ± SD; *n* = 3. (**H**) *N. benthamiana* leaves were separately infiltrated with the indicated *A. tumefaciens* suspensions to detect the function of nucleus-localized FsMEP1 in manipulating plant immune responses. The cell death areas of the indicated infiltrated regions were calculated at 5 d post-inoculation. Values are the means ± SD; *n* = 12. (**I**) The translation efficiency of the indicated proteins was determined by Western blotting using HA antibody. Coomassie bright blue-stained Rubisco protein is shown as the total protein loading control. Statistical analyses were performed using Student’s *t* test; * and ** represent significant differences at *p* < 0.05 and *p* < 0.01.

For a more particular knowledge of the function of FsMEP1 in the mutual effect between the fungus and host, we used *A. tumefaciens*-mediated transformation to express FsMEP1 or ΔSPFsMEP1 (without the signal peptide) instantaneously in *N. benthamiana leaves*. The coding sequences of *FsMEP1*, *ΔSPFsMEP1*, *GFP*, and BAX (Bcl-2-associated X protein) were cloned separately and inserted into the PVX vector pGR107, which includes a C-terminal 3×HA tag. The transient expressions of these genes, as illustrated in Figure 4A, demonstrated that both FsMEP1 and ΔSPFsMEP1 effectively inhibited the cell death induced by BAX in the *N. benthamiana* leaves (Figure 4A). Notably, this suppressive function remained intact even in the absence of the signal peptide (Figure 4A). The immunoblot analysis confirmed that the indicated proteins were efficiently produced with the expected size (Figure 4B). As expected, the host immunity responses represented by reactive oxygen species (ROS) explosion, callose accumulation, and the upregulation of defense-related genes were significantly reduced in the *N. benthamiana* leaves that co-expressed FsMEP1/ΔSPFsMEP1 with BAX as compared to those of BAX (Figure 4C–G). Additionally, the *N. benthamiana* leaves that expressed FsMEP1 and ΔSPFsMEP1 showed enhanced susceptibility to *Botrytis cinereal*, as evidenced by the larger lesion areas in the leaves (Figure S3A). Moreover, we generated a modified FsMEP1 protein that fused with an N-terminus NES sequence (NES-FsMEP1) to determine whether the inhibitory effect of FsMEP1 depended on its subcellular localization. The green fluorescence signal of NES-FsMEP1 nearly disappeared from the nucleus and mainly accumulated in the cytoplasm (Figure S3B). Moreover, the cell death-inhibitory function of NES-FsMEP1 was also abolished (Figure 4H), and the *N. benthamiana* leaves that expressed NES-FsMEP1 showed a similar susceptibility to *B. cinereal* to that of the GFP, as there were no significant differences in the lesion areas in the leaves (Figure S3C). The Western blotting assay confirmed that the indicated proteins were accurately produced with the expected size (Figure 4I). Together, these results indicated that FsMEP1 could effectively inhibit plant immunity responses, and that this is mainly dependent on its *localization* in the host nuclei.

### 2.5. FsMEP1 Interacts with the Thiamine Thiazole Synthase ScTHI2 of Sugarcane

**Figure 5:**
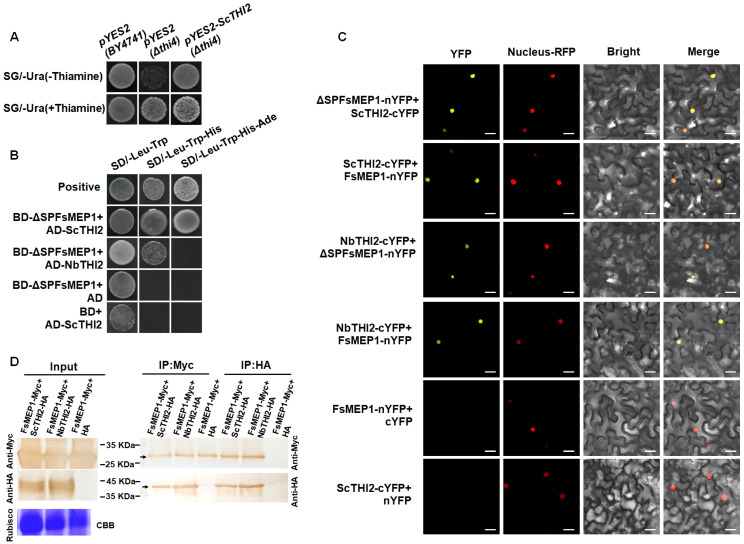
FsMEP1 directly interacts with the thiamine thiazole synthase ScTHI2/NbTHI2. (**A**) Functional complementation of the *S. cerevisiae* thi4 mutant strain BY4741 with ScTHI2. The thi4 mutant strain BY4741 was transformed with *pYES2-FsTHI2*, and the transformed cells were cultured on the SG/-Ura medium without thiamine to confirm the function of FsTHI2. The thi4 mutant strain BY4741 transformed with the empty pYES2 vector served as the negative control. The BY4741 transformed with the empty pYES2 vector was used as the positive control. The yeast cells were grown on SG/-Ura medium with (50 mg/L thiamine) or without thiamine at 30 °C for 3 days. (**B**) Interaction of ΔSPFsMEP1/ScTHI2 and ΔSPFsMEP1/NbTHI2 in yeast two-hybrid (Y2H) assay. Transformed yeast cells were grown on SD media, and the colonies grown normally on SD-Trp-Leu-His and SD-Trp-Leu-His-Ade media indicate positive interactions. (**C**) BiFC assay showing the interactions between ΔSPFsMEP1-nYFP and ScTHI2-cYFP, FsMEP1-nYFP and ScTHI2-cYFP, ΔSPFsMEP1-nYFP and NbTHI2-cYFP, and FsMEP1-nYFP and NbTHI2-cYFP, and that these interaction complexes formed functional YFP in the nuclei. Nucleus–RFP (AtHY5-RFP) served as an RFP marker for nuclei. Merge: merging of YFP and RFP. (**D**) CO-IP analysis for interaction between FsMEP1-Myc and ScTHI2-HA and between FsMEP1-Myc and NbTHI2-HA in vivo. FsMEP1-Myc/ScTHI2-HA and FsMEP1-Myc/NbTHI2-HA were co-expressed in *N. benthamiana* leaves, and total proteins were immunoprecipitations with anti-MYC beads and anti-HA beads. Protein samples and IP products were detected in Western blots with anti-Myc or anti-HA antibody. Bar: 20 μm.

To further identify the host interaction protein of FsMEP1, we screened a yeast two-hybrid (Y2H) library of sugarcane and identified a potential interacting protein named thiamine thiazole synthase ScTHI2. THI4 from *Saccharomyces cerevisiae* (ScTHI4) has been reported to be involved in thiamine synthesis [31], and ScTHI2 showed 53.26% similarity with ScTHI4. To confirm the function of ScTHI2, a thiamine-deficient yeast strain (*Δthi4*) was generated by knocking out the thiamine thiazole synthase gene *THI4* in the yeast strain BY4741, and the expression vector of *S. cerevisiae* (pYES2) that carries ScTHI2 was introduced into the *THI4* knockout mutant strain (*Δthi4*) for functional complementation. The results showed that the *Δthi4* transformed with the pYES2 empty vector grew abnormally in the medium that lacked thiamine, whereas the *Δthi4* transformed with pYES2-ScTHI2 grew as well as the wild type (Figure 5A). The results suggest that ScTHI2 may have a similar function to ScTHI4 in thiamine biosynthesis. Further, the 1:1 Y2H assays showed that the ΔSPFsMEP1 protein showed a strong interaction with ScTHI2 (Figure 5B). Moreover, ΔSPFsMEP1 can also interact with NbTHI2, which showed 75% similarity with ScTHI2, proving that the interaction between FsMEP1 and thiamine thiazole synthase is conserved in plants (Figure 5B). To further confirm the interactions between FsMEP1 and thiamine thiazole synthase in vivo, bimolecular fluorescence complementation (BiFC) and co-immunoprecipitation (Co-IP) were performed, as indicated in Figure 5C,D. The BiFC assay revealed that the interaction between FsMEP1 and ScTHI2/NbTHI2 occurred in the nuclei (Figure 5C), and FsMEP1-MYC, but not MYC alone, precipitated ScTHI2-HA/NbTHI2-HA in the *N. benthamiana* leaves. Similarly, ScTHI2-HA/NbTHI2-HA, but not HA alone, precipitated FsMEP1-MYC in the *N. benthamiana* leaves (Figure 5D).

### 2.6. FsMEP1 Contributes to Virulence by Disturbing Localization of ScTHI2

**Figure 6:**
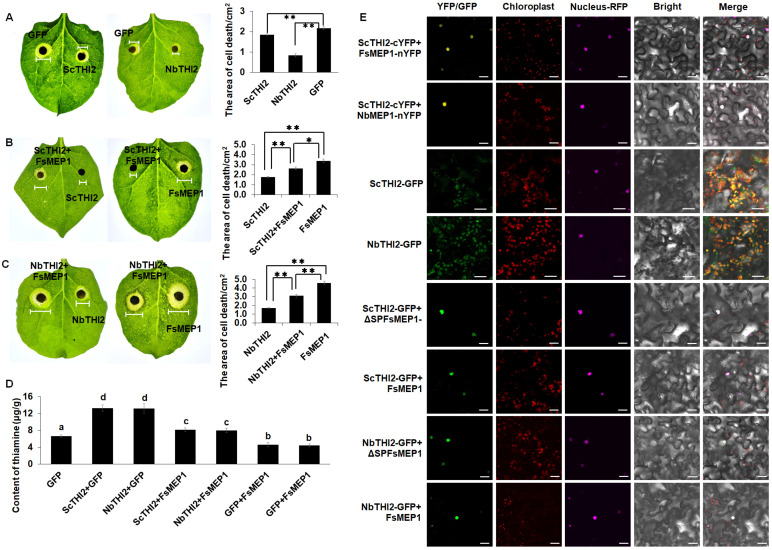
FsMEP1 exerted its host immunity suppression function by directing the localization of ScTHI2 and NbTHI2 to nuclei. (**A**) Transient expressions of ScTHI2 or NbTHI2 enhanced *N. benthamiana* resistance to *B. cinerea*. Representative photographs were taken at 3 d post-*B. cinerea*-inoculation. Statistical analysis of the lesion area caused by *B. cinerea* in *N. benthamiana* leaves transiently expressing the indicated constructs was performed at 3 d post-*B. cinerea*-inoculation. Values are the means ± SD; *n* = 6. (**B**,**C**) The presence of FsMEP1 attenuated ScTHI2 and NbTHI2-mediated resistance to *B. cinerea*. (**D**) Representative photographs were taken at 3 d post-*B. cinerea*-inoculation. Statistical analysis of the lesion area caused by *B. cinerea* in *N. benthamiana* leaves transiently expressing the indicated constructs was performed at 3 d post-*B. cinerea*-inoculation. Values are the means ± SD; *n* = 6. (**D**) The thiamine content in the *N. benthamiana* leaves that were inoculated with the indicated constructs for 5 days. Values are the means ± SD; *n* = 9. (**E**) FsMEP1 directed the chloroplast-localized ScTHI2 and NbTHI2 to nuclei to inhibit their function in thiamine biosynthesis. Bar: 20 μm. Statistical analyses were performed using Student’s *t* test; * and ** represent significant differences at *p* < 0.05 and *p* < 0.01; different letters represent significant differences at *p* < 0.05.

Given the importance of thiamine thiazole synthase in plant immune regulation, we focused on the molecular mechanisms by which FsMEP1 interacts with ScTHI2/NbTHI2 to suppress plant immunity. The transient expressions of ScTHI2 and NbTHI2 were detected in the *N. benthamiana* leaves, which were subsequently inoculated with *B. cinerea*. The experimental results showed that ScTHI2 and NbTHI2 could efficiently improve the plant resistance to *B. cinerea*, as evidenced by the smaller lesion areas in the *N. benthamiana* leaves transiently expressing ScTHI2 or NbTHI2 than those of the GFP (Figure 6A), suggesting that *ScTHI2* and *NbTHI2* are positive regulators of plant immunity. The effects of FsMEP1 on ScTHI2/NbTHI2 were further confirmed by the co-expression of FsMEP1 with ScTHI2 or NbTHI2 in the *N. benthamiana* leaves subsequently inoculated with *B. cinerea*. As anticipated, the presence of FsMEP1 significantly compromised the defense response to *B. cinerea* induced by ScTHI2 or NbTHI2. This was evidenced by the larger lesion areas observed in the *N. benthamiana* leaves co-expressing FsMEP1 with ScTHI2 or NbTHI2 compared to those expressing only ScTHI2 or NbTHI2 (Figure 6B,C). Moreover, the thiamine content in the inoculated *N. benthamiana* leaves also significantly weakened in the present of FsMEP1 (Figure 6D). These results proved that FsMEP1 significantly enhanced the host susceptibility by interacting with ScTHI2 or NbTHI2. The existing studies have shown that THI1/THI2 is a multifunctional protein that functions in repairing DNA damage when targeting mitochondria and is involved in thiamine synthesis when targeting chloroplasts [33]. Our results demonstrated that FsMEP1 is a nucleus-localization protein and that it interacted with ScTHI2 and NbTHI2 both in vitro and in vivo, and the FsMEP1-THI2s interaction complexes were also localized in the nucleus. Therefore, it is necessary to investigate whether FsMEP1 blocks thiamine synthesis and THI2-mediated disease resistance by disturbing the normal subcellular localization of THI2. As illustrated in Figure 6E, the expressions of ScTHI2-GFP and NbTHI2-GFP were observed within chloroplasts; however, in the presence of FsMEP1, both ScTHI2-GFP and NbTHI2-GFP were detected in the nucleus. Taken together, FsMEP1 could suppress plant immunity and contribute to the pathogenicity of *F. sacchari* by disturbing the normal localization of ScTHI2 and NbTHI2, resulting in impaired thiamine biosynthesis and THI2-mediated defense responses.

## 3. Discussion

Since the inception of plant–pathogen interactions, a fierce but silent game has been played between pathogens and plants [34]. Unlike mammals, plants are sessile and lack mobile defense cells and somatic adaptive immune systems [35]. However, in the long course of evolution, to protect themselves from pathogens, plants have evolved an innate immune system to sense, recognize, and distinguish the systemic signals emanating from pathogen–infection sites and promote the corresponding defensive responses. Initially, the PAMPs are recognized by the plasma membrane-localized PRRs to launch the PTI to impede pathogens’ invasion and colonization [36]. In the subsequent phase, pathogens employ effectors to manipulate the PTI, ultimately resulting in effector-triggered susceptibility (ETS). In response, plants deploy NLR protein receptors to detect these effectors, thereby initiating a second layer of their immune response known as effector-triggered immunity (ETI) [37]. Numerous studies have demonstrated that the effector proteins produced by pathogens are significant factors contributing to plant infestations and function at the interfaces of diverse interactions between pathogens and hosts to facilitate the pathogenicity [38,39]. As a result, effector proteins are being increasingly recognized as valuable tools for enhancing the identification and management of resistance genes in modern breeding practices.

Once secreted by pathogens, the effectors target different parts of the host cell and can be roughly categorized as apoplastic and cytoplasmic effectors [40]. At present, the apoplastic effectors that have been identified are mainly plant cell wall-degrading enzymes (CWDEs), which function in disrupting the chemical and physical barriers of the host’s first defense system [41]. The cytoplasmic/intracellular effectors target numerous subcellular organelles, including the plasma membrane, nucleus, cytoplasm, chloroplast, mitochondria, and so on, to interfere with the essential immune components to facilitate pathogen infection [42,43,44]. Hence, in a certain sense, the accurate subcellular localization determines the ability of effectors to function in suppressing plant immunity. Furthermore, different subcellular localizations also indicate variations in their working mechanisms. The research on metalloproteinases as effector proteins has primarily focused on animals–pathogens and their regulation in human-related diseases such as emphysema, achalasia, and cancer, but there have been few reports on effector proteins related to plants–pathogens [45,46,47]. One of the best studied is Avr-Pita, a secreted zinc metalloprotease from *Magnaporthe oryzae* that can be recognized by the leucine-rich repeat domain protein Pi-ta [48]; however, little is known about how and where *Avr-Pita* functions in manipulating host immunity. A recent study demonstrated that Avr-Pita targeted host mitochondria and directly interacted with a mitochondrial cytochrome *c* oxidase (COX) assemble protein (OsCOX11) of the mitochondrial electron transport chain to promote the COX activity in ROS metabolism, thereby inhibiting the ROS explosion and suppressing the congenital immunity in rice upon infection with *M. oryzae* [49]. Another example of a secreted M35 metalloprotease effector is FocM35_1 from *Fusarium oxysporum*, which serves as an essential virulence effector for *Foc TR4*. Further analyses revealed that FocM35_1 was significantly upregulated during the early stages of *Foc TR4* infection progression and could directly interact with the banana chitinase MaChiA in the plasma membrane to inhibit its chitinase activity. Consequently, this interaction led to the suppression of banana immunity [50]. The above studies on metalloprotease-type effectors from plant pathogens have confirmed their roles in virulence and their localization to different subcellular organelles to play roles in manipulating host immunity. Here, we identified a secreted zinc metalloprotease from *F. sacchari*, designated FsMEP1, as a typical effector protein, which was essential for the complete virulence of *F. sacchari* and could suppress the host immune responses (Figure 2, Figure 3 and Figure 4). Further research showed that when the NES sequence was fused to the N-terminus of FsMEP1, its green fluorescence signal mainly disappeared from the nuclei but accumulated near the plasma membrane and cytoplasm. Meanwhile, its cell death-inhibitory function was significantly impaired, which suggests that FsMEP1 should target the host nuclei to effectively inhibit plant immunity responses (Figure S3 and Figure 4), and which proves a novel localization of metalloproteases in pathogen–host interactions. There is increasing evidence that some pathogen effectors hijack the host importin-α that mediates the nuclear import system to target plant nuclei by the nuclear localization signals (NLSs) within the protein [51,52,53]. The translocation of effector proteins into the nucleus has also been observed in certain cases [54]. There was no NLS sequence predicted and identified in the FsMEP1 protein, so how the MEP1 protein is transported to the nucleus requires further investigation.

To understand how FsMEP1 regulates host immunity, yeast double-hybrid assays were implemented with ΔSPFsMEP1 (without the signal peptide) as bait against a cDNA library from sugarcane. The thiamine thiazole synthase ScTHI2 was identified as a clear target of ΔSPFsMEP1 (Figure 5A,B). At the same time, *NbTHI2*, a homolog of *ScTHI2* in *N. benthamiana*, was cloned, and 1:1 Y2H, BiFC, and Co-IP assays between ΔSPFsMEP1/ FsMEP1 and ScTHI2 and ΔSPFsMEP1/ FsMEP1 and NbTHI2 were performed and further confirmed the interactions in the nucleus. ScTHI2 and NbTHI2 have been demonstrated to act as positive regulators of plant resistance against *B. cinerea*, as evidenced by the smaller lesion areas observed in the *N. benthamiana* leaves that transiently express ScTHI2 or NbTHI2 (Figure 6A,B). Furthermore, in the presence of FsMEP1, the defense response to *B. cinerea* induced by ScTHI2 or NbTHI2 was significantly compromised (Figure 6C), and the thiamine contents in the inoculated *N. benthamiana* leaves were also significantly reduced in the presence of FsMEP1 (Figure 6D), suggesting that FsMEP1 could suppress the immunity responses and thiamin biosynthesis that are mediated by ScTHI2 or NbTHI2. Further experiments demonstrated that in the presence of FsMEP1, ScTHI2 and NbTHI2 were detected in the nuclei rather than chloroplasts, thereby resulting in the impaired thiamine biosynthesis and THI2-mediated defense responses (Figure 6E). VB1 is a critical component in several enzymatic reactions for plants, and numerous studied have shown that thiamine could protect plants successfully against a broad spectrum of causative agents [55,56,57]. THI1/THI2 are essential enzymes in thiamine biosynthesis, and studies have revealed that THI1/THI2 is a multifunctional protein involved in DNA damage repair when localized in mitochondria and participates in thiamin synthesis when localized in chloroplasts [31,33]. Notably, a recent study on wheat showed that the Chinese wheat mosaic virus (CWMV) could direct the chloroplast-localized TaTHI1 to the wheat cell membrane, and the overexpression of *TaTHI2* in the wheat conferred enhanced resistance to CWMV with accumulated ROS explosion production by directly suppressing TaCPK5-activated TaCAT1 [31]. This research revealed a novel function of THI1/THI2 in plant immunity through its direct interaction with other proteins to manipulate the defense-related molecule(s). Although, in this work, we provide conclusive evidence for the conclusion that FsMEP1 contributes to virulence by disturbing the localization of ScTHI2/NbTHI2, the mechanism(s) of ScTHI2/NbTHI2-mediated defense responses remain largely unknown.

**Figure 7:**
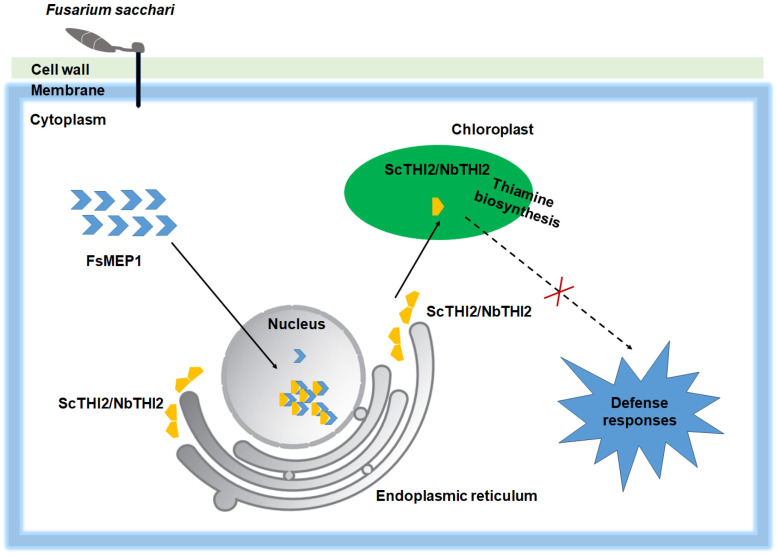
Proposed working model illustrating FsMEP1 and THI2 module in plant–pathogen interactions. FsMEP1 contributes to virulence by disturbing the localization of ScTHI2/NbTHI2.

In conclusion, we propose a model in which FsMEP1-ScTHI2/NbTHI2 plays a pivotal role in manipulating plant immunity (Figure 7). FsMEP1 may facilitate the translocation of chloroplast-localized ScTHI2/NbTHI2 to the nuclei. Abnormalities in the localization of ScTHI2/NbTHI2 lead to impaired thiamine biosynthesis and disrupted THI2-mediated defense responses. Our findings unveil a novel working model for pathogen metalloproteases in host–pathogen interactions. Furthermore, FsMEP1 and ScTHI2 represent valuable genes that can be utilized in genetic engineering approaches, such as host-induced gene silencing (HIGS) targeting FsMEP1 and the manipulation of endogenous VBI synthesis through the overexpression of ScTHI2, to enhance the sugarcane resistance to *F. sacchari*.

## 4. Materials and Methods

### 4.1. Cultivation of Microbial Strains and Plant Materials

The *Fusarium sacchari* isolate CNO-1 was cultured on potato dextrose agar (PDA) media. *Agrobacterium tumefaciens* GV3101 and AGL1 were employed for the *Agrobacterium*-mediated expression experiments in *Nicotiana benthamiana* and fungal transformations, respectively. The *F. sacchari* knockout mutants and complementary mutants were cultured on PDA with 50 µg/mL hygromycin and 50 µg/mL geneticin, respectively. *Botrytis cinerea* strain B05.10 was cultured on PDA at 25 °C for disease resistance analysis. The *F. sacchari*-susceptible sugarcane cultivar ZZ1 (modern hybrid sugarcane cultivar Zhongzhe No. 1) was grown in a greenhouse maintained at 28 °C, a 14 h/10 h light/dark photoperiod, and 65  ±  5% relative humidity, and the six-leaf-stage sugarcane plants were inoculated with *F. sacchari* for disease resistance analysis, as described previously [32]. *N. benthamiana plants* were grown in soil in a growth chamber maintained at 28 °C, a 14 h/10 h light/dark photoperiod, and 65  ±  5% relative humidity.

### 4.2. Identification and Phylogenetic Analysis of FsMEP1

The candidate extracellular protein with the typical zinc-dependent metalloprotease domain FsMEP1 was identified and cloned from *F. sacchari* strain CNO-1. SignalP v. 6.0 (D-score cut-off set to 0.500, https://services.healthtech.dtu.dk/services/SignalP-6.0/, accessed on 28 March 2024) was used to identify the signal peptide and cleavage site of FsMEP1. The phylogenetic analysis of FsMEP1 and other MEP proteins was constructed using the neighbor-joining method in MEGA11 software. DNAMAN V6.0 software was used for multiple sequence alignment.

### 4.3. RNA Extraction and RT-Quantitative PCR Analysis

RNA was extracted using the Eastep^®^ Super Total RNA Extraction Kit (Promega) following the manufacturer’s instructions. First-strand cDNA was generated from 3 μg of total RNA using the HiScript II Reverse Transcriptase Kit (Vazyme) according to the manufacturer’s instructions [58]. RT-qPCR was performed on a LightCycle^TM^ 96 Real Time PCR system (Roche) in a 15 μL reaction volume that consisted of 7.5 μL ChamQ SYBR Color qPCR Master Mix (Vazyme, Nanjing, China), 0.25 μL 10 μM forward/reverse primers, and 7.0 μL cDNA, following the manufacturer’s instructions. *FvActin* and *NbEF1* were provided as reference genes for the sugarcane and *N. benthamiana*, respectively. All RT-qPCR experiments were set up in three biological replicates, and each biological replicate experiment contained three technical replicates. The 2^−ΔΔCT^ method was used to assess the relative expression levels of the target genes [59]. All primers for RT-qPCR are listed in Table S1.

### 4.4. Yeast Signal Sequence Trap System

The function of the predicted signal peptide of FsMEP1 was verified using the yeast signal sequence trap system as described previously [60]. The coding sequence of the predicted signal peptide of *FsMEP1* was cloned and fused in frame in the *pSUC2* vector at the *EcoR*I-*Xho*I sites. The recombinant construct *SPFsMEP1-pSUC2* and the indicated control constructs (negative controls: empty *pSUC2* vector and *SPMg87-pSUC2*; positive control: *SPAvr1b-pSUC2*) were implanted into the yeast strain YTK12. The positive inverters were cultured on CMD-W (free from tryptophan) medium and YPRAA medium containing 2% raffinose for invertase secretion detection. The invertase activities of all the yeast strains were further determined by testing the TTC reduction to the insoluble red product triphenyl formamide.

### 4.5. Subcellular Localization Assays

The coding sequences of *FsMEP1*, *ScTHI2*, *NbTHI2*, and *ΔSPFsMEP1* (lacking the region encoding the signal peptide) were amplified and inserted into the pGWB418-GFP vector at the *Afe* I site to generate the C-terminus-fused GFP constructs. The coding sequences of *FsMEP1* and *ΔSPFsMEP1* were also amplified and impaled into the pCambia1300-NES-GFP vector at the *Kpn* I-*Bam*H I sites to generate the N-terminus-fused nuclear export signal (NES) and C-terminal-fused GFP constructs. *AtHR5-RFP* (nucleus–RFP) served as a nuclear marker. All vectors were transformed into *N. benthamiana* plants for transient expression via the *A. tumefaciens* strain GV3101. Fluorescence signals in leaf epidermal cells were observed using a confocal microscope (Olympus FV3000) at 60 h post-infiltration. The primers used in this study are enumerated in Table S1.

### 4.6. F. sacchari Transformations for Gene Deletion and Complementation

To generate *FsMEP1* knockout and complementary mutants in *F. sacchari*, the *Agrobacterium*-mediated homologous recombination-based knockout and complementation technique was employed, and the positive transformants were detected by PCR with the appropriate test primer pairs. Approximately 1000 bp 5′ and 3′ regions of the targeted gene *FsMEP1* were amplified from the DNA of *F. sacchari* and inserted into a pGKO-Hyg vector that carries hygromycin B resistance at the *Pac* I site using the ClonExpress Ultra One Step Cloning Kit V2 (Vazyme, Nanjing, China) to generate the *FsMEP1* deletion construct. The sequence, including the 847 bp 5′ region, *FsMEP1* sequence, and 738 bp 3′ regions, were received from the DNA of *F. sacchari* and inserted into the pDHtsk-G418 vector that carries geneticin resistance at the *Hind* III and *Bam*H I sites to generate the *FsMEP1* complementation construct. The positive recombinant constructs were transferred into *A. tumefaciens* strain *AGL1* and the fungal transformation into the wild-type *F. sacchari* strain CNO-1 for gene deletion, and into the positive *FsMEP1* knockout mutant strain for *FsMEP1* complementation, which was performed as described previously [61]. The positive transformants were screened on the PDA medium with 50 µg/mL hygromycin B or 50 µg/mL geneticin for gene deletion and complementation, respectively. The primers used in this study are enumerated in Table S1.

In order to examine the impact of FsMEP1 in the mycelial growth and morphology of *F. sacchari*, 10^5^ conidia/mL suspensions of wide-type CNO-1, *FsMEP1* knockout mutants, and *FsMEP1* complementary mutants were prepared and inoculated at the center of the medium with different kinds of carbon sources. The colony morphology, growth rate, conidia morphology, and conidia production were recorded at 3 d, 6 d, and 9 d post-inoculation.

### 4.7. Pathogenicity Assays

The pathogenicity assays of the *FsMEP1* knockout mutants and complementary mutants were conducted on sugarcane leaves as described previously [62]. The wild-type CNO-1, *FsMEP1* knockout mutants, and *FsMEP1* complementary mutants were pre-cultured on the PDA medium for 7 days, and the colonized agar plugs were inoculated on the leaves from the six-leaf-stage seedling of ZZ1. The necrotic areas of the inoculated leaves were gauged at 7 d post-inoculation, and, simultaneously, the leaf samples were collected, sterilized, and inoculated on PDA medium for fungal recovery assays.

### 4.8. Agrobacterium-Mediated Transient Expression in N. benthamiana

To determine the functions of specific domains of FsMEP1 in manipulating host immune responses, the sequences of *FsMEP1*, *ΔSPFsMEP1*, *NES*-*ΔSPFsMEP1* (5′ fused the nuclear export signal sequence), *GFP*, and *BAX* (BCL-2 associated X) were amplified and cloned into the PVX vector pGR107 with a C-terminal 3×HA tag. All the recombinant constructs were transformed into the *A. tumefaciens* strain and infiltrated into the leaves of 4- to 5-week-old *N. benthamiana* plants using a 1 mL needleless syringe, as described previously [63]. The BAX and GFP were used as positive and negative controls, respectively. The cell death symptoms of the inoculated *N. benthamiana* leaves were recorded from 3 to 7 days during the experiment.

To verify the translation of the indicated proteins in the inoculated *N. benthamiana* leaves, the total protein was extracted using the RIPA lysis buffer (Thermo Scientific) from the infiltrated *N. benthamiana* leaves 48 h after vaccination and purified with Pierce Anti-HA magnetic beads (Thermo Scientific) following the manufacturer’s instructions. Next, 10 μL protein samples were subjected to 12% SDS-PAGE and immunoblotted with anti-HA antibody (ABclonal, Wuhan, China) and the Enhanced HRP-DAB Chromogenic Substrate Kit (Tiangen, Beijing, China) to determine the protein levels.

### 4.9. Botrytis Cinereal Inoculation Assay

Target proteins were expressed in *N. benthamiana* leaves via agroinfiltration, as described above, and the *Botrytis cinereal* strain B05.10 was cultured on PDA at 25 °C for 7 days. Five-millimeter-wide mycelia plugs were placed in the infiltrated regions of the *N. benthamiana* leaves at 24 h post-infiltration. Inoculated samples were placed in a big plate with a shallow nutrient solution (1/2 MS) and kept in a growth chamber at 80% humidity under a 14 h/10 h light/dark photoperiod. Lesion areas were recorded and photographed from 3 to 5 days during the experiment.

### 4.10. Determination of ROS Burst and Callose

The ROS staining and callose staining in *N. benthamiana* leaves were examined using the 3, 3′-diaminobenzidine (DAB) staining method and aniline blue staining method, as described previously [41]. The measurement of H_2_O_2_ was performed using the Micro Hydrogen Peroxide Assay Kit (Solarbio, Beijing, China) following the manufacturer’s instructions.

### 4.11. Y2H Assays

A cDNA library derived from sugarcane leaves was previously constructed in the yeast vector pGADT7 using Matchmaker Library Construction and Screening Kits. The coding sequence of *ΔSPFsMEP1* was amplified and cloned into pGBKT7 to generate the bait vector *BD-ΔSPFsMEP1* at the *Eco*R I-*Bam*H I sites and transformed into yeast strain Y2HGold for library screening. The positive clones were selected on SD/-Leu/-Trp/-His and SD/-Leu/-Trp/-His/-Ade media. The gene sequences of the candidate interaction proteins were verified by individual AD plasmid extraction and sequencing. We performed 1:1 Y2H experiments to further validate the interaction in yeast cells. The coding sequences of *ScTHI2* and *NbTHI2* were amplified from the cDNA of sugarcane and *N. benthamiana*, respectively, and inserted into pGADT7 at the *Eco*R I-*Bam*H I sites to generate *AD- ScTHI2* and *AD-NbTHI2*. *AD-ScTHI2* and *BD-ΔSPFsMEP1* and *AD-NbTHI2* and *BD-ΔSPFsMEP1* were co-transformed into the yeast strain Y2HGold. The co-transformed cells were cultured on SD/-Leu/-Trp, SD/-Leu/-Trp/-His, and SD/-Leu/-Trp/-His/-Ade media for 3–5 days at 30 °C. Yeast cells co-transformed with *BD-ΔSPFsMEP1* and empty *pGADT7*, empty *pGBKT7*, and *AD-ScTHI2* were used as a negative control [64].

### 4.12. BiFC and Co-IP Assays

To generate the BiFC constructs, the coding sequences of *FsMEP1*, *ΔSPFsMEP1*, *ScTHI2*, and *NbTHI2* were amplified and subsequently cloned into the BiFC vectors pS1301-nYFP (containing a C-terminal MYC tag) or pS1301-cYFP (containing a C-terminal HA tag) at the *Xba*I-*BamH*I restriction sites, resulting in the creation of the constructs *FsMEP1-nYFP*, *ΔSPFsMEP1-nYFP*, *ScTHI2-cYFP*, and *NbTHI2-cYFP*, respectively [65]. The recombinant plasmids were individually transformed into GV3101 strains and infiltrated into the leaves of four- to five-week-old *N. benthamiana* plants, as outlined in the BiFC assays, using a 1 mL needleless syringe. Fluorescence signals within leaf epidermal cells were observed utilizing a confocal microscope (Olympus FV3000, Tokyo, Japan) at 60 h post-infiltration. The primers employed in this study are detailed in Table S1.

The Co-IP assay was performed according to a previous report with minor modifications [66]. In brief, the *N. benthamiana* leaves that showed intense fluorescence of YFP (co-infiltrated with ScTHI2-cYFP/FsMEP1-nYFP and NbTHI2-cYFP/FsMEP1-nYFP) and the negative control (co-infiltrated with cYFP/FsMEP1-nYFP) in the BiFC assays were collected and ground into powder in liquid nitrogen and homogenized in 1 mL of RIPA lysis buffer (50 mM Tris-HCl pH 7.5, 150 mM NaCl, 1 mM EDTA, 1 mM DTT, 1% [*v*/*v*] IGEPAL CA-630, 10% [*v*/*v*] glycerol, 1 mM PMSF, and protease inhibitor cocktail). Immunoblotting and Co-IP experiments were performed following instructions of the Pierce Magnetic c-Myc-Tag IP/Co-IP Kit (Thermo Scientific) and Pierce Magnetic HA-Tag IP/Co-IP Kit with HA and Myc antibodies (ABclonal, Wuhan, China), using the Enhanced HRP-DAB Chromogenic Substrate Kit (Tiangen, Beijing, China) to visualize the result.

### 4.13. Detection of Thiamine Content

The detection of the *thiamine content* in the *N. benthamiana* leaves was conducted as previously described [31]. Briefly, approximately 1 g of leaf samples was ground in liquid nitrogen and homogenized in 5 mL of a reagent mixture (0.1 mol/L hydrochloric acid: methanol, 20:80 *v*/*v*). The mixture was then subjected to ultrasonic extraction at room temperature for 60 min. Following centrifugation at 12,000 rpm for 20 min at 4 °C, the supernatant was collected and filtered through a 0.45 μm filter before being analyzed by HPLC.

### 4.14. Accession Numbers

Sequence data from this article can be found in the GenBank databases under the following accession numbers: ScTHI2, YZ-Rec-Chr02A0039120 (the genomic sequences of ZZ1 were obtained from the China National Center for Bioinformation Genome Warehouse under accession code GWHEQVP00000000, https://ngdc.cncb.ac.cn/gwh/Assembly/83532/show, accessed on 13 September 2024); NbTHI2, XM_019397805.1; Saccharomyces cerevisiae ScTHI4, NM_001181273.1; NbPR1, OQ675540.1; NbPR2, XM_019376146.1; NbHSR203J, NW_015819496.1; NbLOX, KC585517.1; FvActin, XM_018889830.1; NbEF1, PQ008965.1.

## Data Availability

All the data that support the findings of this study are available in the paper and Supplementary Materials.

## Supplementary Materials

The following supporting information can be downloaded at https://www.mdpi.com/article/10.3390/ijms252212075/s1.
