# Supplementary material for: Fusarium sacchari Effector FsMEP1 Contributes to Virulence by Disturbing Localization of Thiamine Thiazole Synthase ScTHI2 from Sugarcane
Source: Int J Mol Sci. 2024 Nov 10;25(22):12075. doi: 10.3390/ijms252212075 (PMC11593444; doi:10.3390/ijms252212075)
Supplement: Supplementary file 1 [file ijms-25-12075-s001.zip › Supplemental figures.pdf]

Supplemental figures

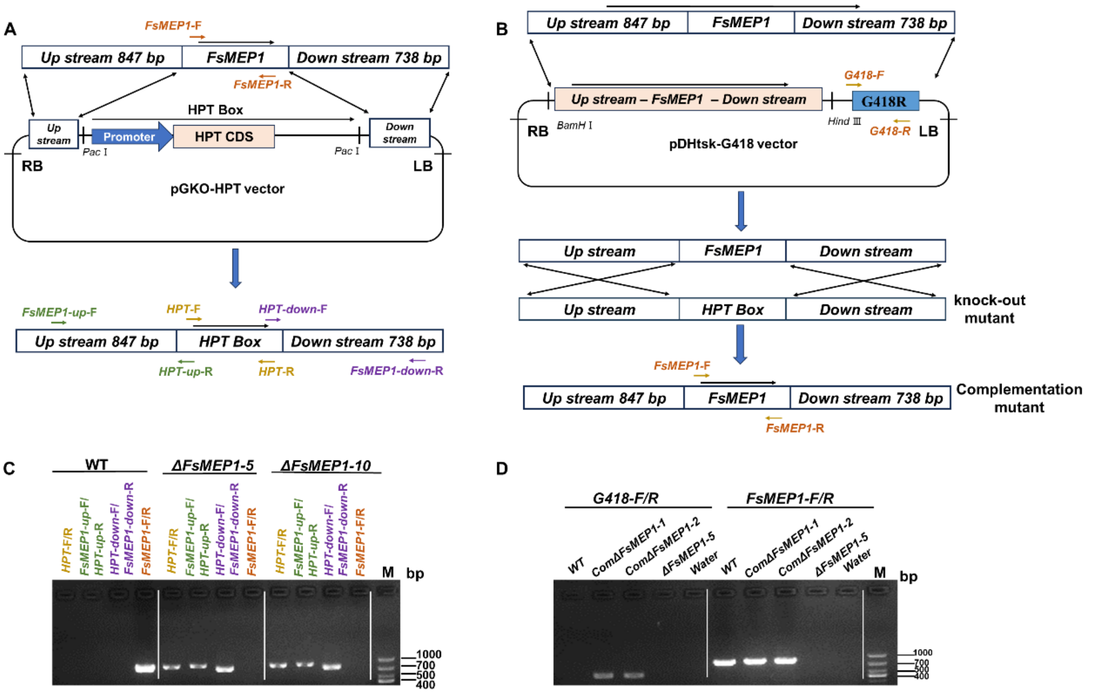

Fig. S1. The generation of *FsMEP1* knockout and complementary *mutants* in *Fusarium sacchari*. (A) Schematic representation of the strategy used to knock out *FsMEP1* in *F. sacchari*. (B) Schematic representation of the strategy used to generate *FsMEP1* complementary mutants in Δ*FsMEP1* strain. (C) PCR identification of the *FsMEP1* knockout mutants (Δ*FsMEP1*) using the primer pairs as indicated in (A). (D) PCR identification of the *FsEG1* complementary mutants (*Com*Δ*FsMEP1*) using the primer pairs as indicated in (B).

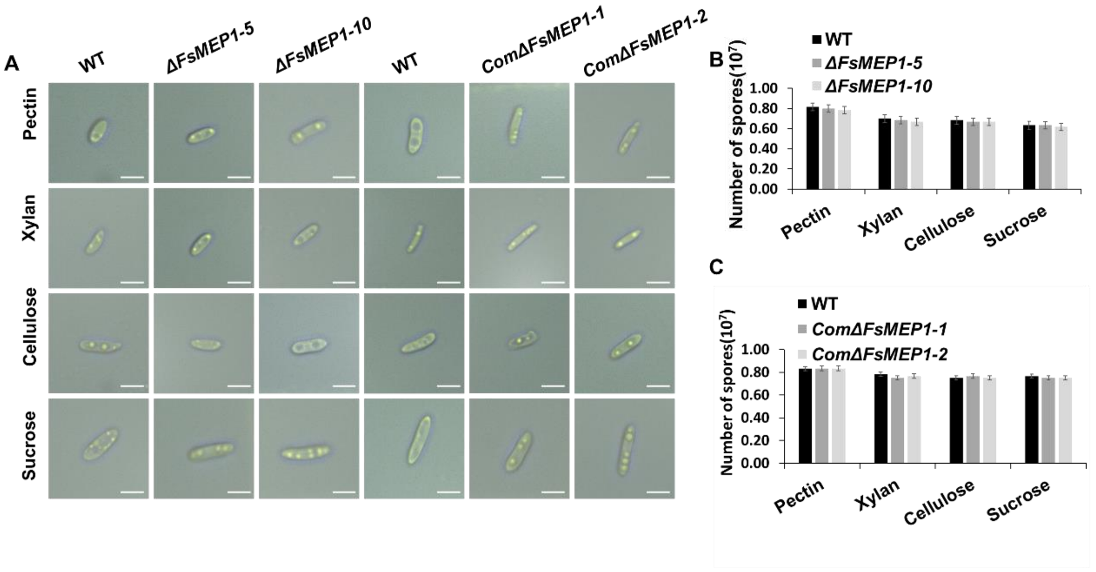

Fig. S2. FsMEP1 from *F. sacchari* was not required for conidia morphology and conidia production. (A) The conidia morphology of ΔFsMEP1*, ComΔFsMEP1* and wild-type strains grown on medium with pectin, xylan, cellulose or sucrose as carbon source for 9 days. (B)-(C) Statistical

analysis of the conidia productions of *ΔFsMEP1*, *ComΔFsMEP1* and wild-type strains grown medium with pectin, xylan, cellulose or sucrose as carbon source. Values are the means ± SD; n = 6. Bar: 10 μm.

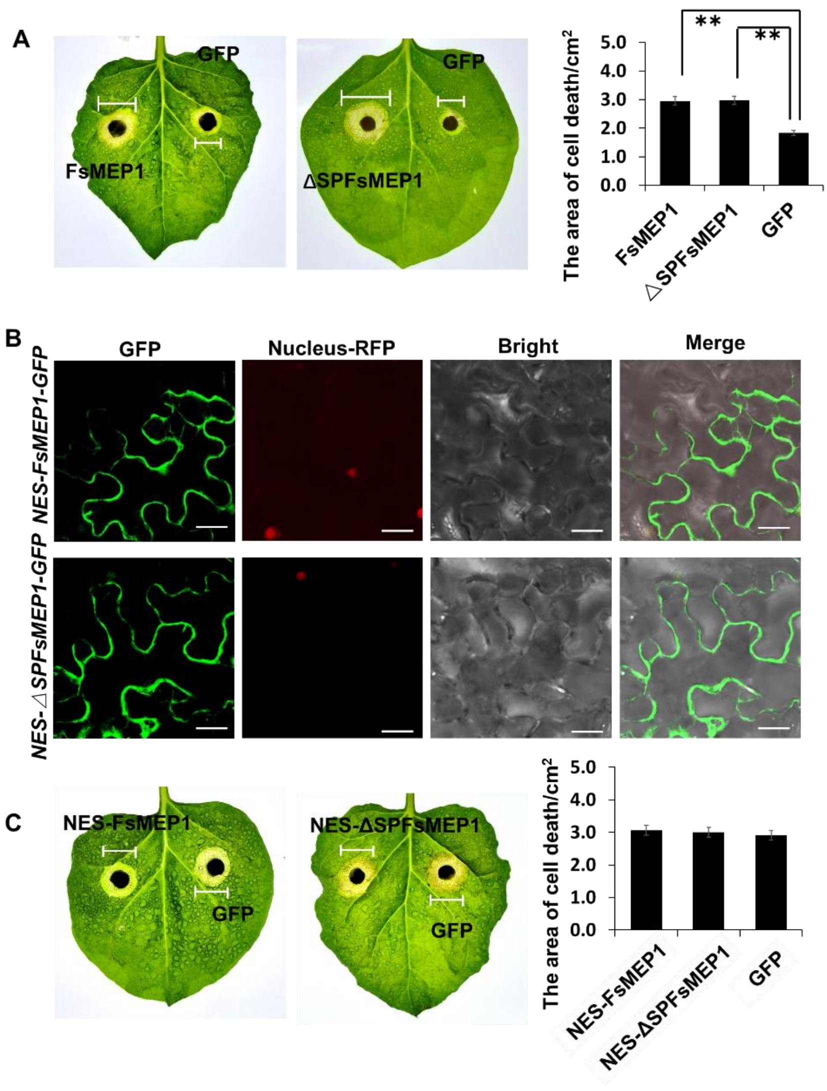

Fig. S3. FsMEP1 localized on the plant nuclei to exert its host immunity suppression function. (A) Transiently expression FsMEP1 or ΔSPFsMEP1 enhanced *N. benthamiana* susceptibility to *B. cinerea.* Representative photographs were taken at 3 d post *B. cinerea* inoculation. Statistical analysis of the lesion area caused by *B. cinerea* in *N. benthamiana* leaves transiently expressing the indicated constructs were taken at 3 d post *B. cinerea* inoculation. Values are the means ± SD; n = 6. (B) Subcellular localization of the FsMEP1 and ΔSPFsMEP1 fused with N-terminus NES and C-terminus GFP. Representative leaves were photographed at 60 h post inoculation. (C) The function of FsMEP1 in inhibiting host immunity was dependent of its localization in the nuclei. Representative photographs were taken at 3 d post *B. cinerea* inoculation. Statistical analysis of the lesion area caused by *B. cinerea* in *N. benthamiana* leaves transiently expressing the indicated constructs were taken at 3 d post *B. cinerea* inoculation. Values are the means ± SD; n = 6. Statistical analyses were performed using Student's *t* test, * and ** represent significant differences at $p<0.05$ and $p<0.01$. Bar: 20 μm.
